# EUS-guided gastroenterostomy for benign gastric outlet obstruction: Clinical and technical outcomes from a multicenter cohort study

**DOI:** 10.1055/a-2778-5386

**Published:** 2026-01-22

**Authors:** Anam Rizvi, Kamal Hassan, Kate Johnson, Mouen A. Khashab, Farimah Fayyaz, Michel Al Mardini, Jayanta Samanta, Jahnvi Dhar, Alberto Larghi, Carlos Robles-Medranda, Juan Alcivar Vasquez, Martha Arevalo, Edoardo Forti, Irene Cottone, Jorge Vargas, Amrita Sethi, Kavel Visrodia, Enad Dawod, Mark Hanscom, Kartik Sampath, Srihari Mahadev, David L. Carr-Locke, Laith Eyad Baqain, Reem Z Sharaiha, Working Group

**Affiliations:** 1 Division of Gastroenterology and Hepatology, Department of Medicine, New York - Presbyterian Weill Cornell Medical Center, New York, United States; 2 1466 Gastroenterology, Johns Hopkins University, Baltimore, United States; 3 Department of Gastroenterology and Hepatology, Postgraduate Institute of Medical Education and Research, Chandigarh, India; 4 18654 Department of Gastroenterology, Fondazione Policlinico Universitario Agostino Gemelli IRCCS, Rome, Italy; 5 Department of Gastroenterology, Instituto Ecuatoriano de Enfermedades Digestivas, Guayaquil, Ecuador; 6 Endoscopy, Omni Hospital, Guayaquil, Ecuador; 7 Digestive Endoscopy, Ospedale Niguarda-Ca' Granda, Milan, Italy; 8 Department of Gastroenterology and Hepatology, Caja Costarricense de Seguro Social, San Jose, Costa Rica; 9 21611 Gastroenterology and Hepatology, Columbia University Medical Center, New York, United States; 10 21611 Digestive and Liver Diseases, Columbia University Irving Medical Center, New York, United States; 11 12295 Division of Gastroenterology and Hepatology, Weill Cornell Medicine, New York, United States; 12 155133 Department of Medicine, King Hussein Medical Center, Amman, Jordan

**Keywords:** Endoscopy Upper GI Tract, Endoscopic resection (ESD, EMRc, ...), Endoscopic ultrasonography, Intervention EUS

## Abstract

**Background and study aims:**

Endoscopic ultrasound-guided gastroenterostomy (EUS-GE) is emerging as a minimally invasive alternative for benign gastric outlet obstruction (GOO). This study evaluated its safety, efficacy, and long-term outcomes.

**Patients and methods:**

This international, multicenter retrospective study included 72 patients who underwent EUS-GE for benign GOO across 13 centers. Clinical success was defined as ability to tolerate solid food based on the GOO scoring system. Secondary outcomes included technical success and adverse events (AEs).

**Results:**

Technical success was achieved in 97% of cases. Ability to tolerate solid food improved significantly, from 4% pre-procedure to 90% at 60 days post-procedure (<i>P</i> < 0.00001). Mean time to oral intake was 20.1 hours. AEs were mild,

occurring in 6% intra-procedurally and 10% post-procedurally. Mean follow-up duration was 426.3 days.

**Conclusions:**

EUS-GE demonstrates high technical and clinical success rates with a favorable safety profile for benign GOO, offering a promising alternative to traditional surgical approaches.

## Abkürzungen

GOO: Gastric Outlet Obstruction
EUS-GE: Endoscopic Ultrasound-Guided Gastroenterostomy
LAMS: Lumen-Apposing Metal Stents
SGJ: Surgical Gastrojejunostomy
EBD: Endoscopic Balloon Dilation
AEs: Adverse Events
ASGE: American Society for Gastrointestinal Endoscopy
OTSC: Over-the-Scope Clip
SD: Standard Deviation
IQR: Interquartile Range

## Introduction

Benign gastric outlet obstruction (GOO), caused by conditions such as peptic ulcer disease, chronic pancreatitis, and Crohn’s disease, leads to debilitating symptoms like nausea, vomiting, and weight loss 1 . Although advances in medical therapy have reduced its incidence, benign GOO still poses a therapeutic challenge.

Traditional treatments like endoscopic balloon dilation (EBD) and surgical gastrojejunostomy (SGJ) have limitations. EBD often requires multiple sessions with a risk of perforation 2 , whereas SGJ, although effective, carries significant morbidity, especially for patients with comorbidities 3 .

Endoscopic ultrasound-guided gastroenterostomy (EUS-GE) has emerged as a minimally invasive alternative, creating a bypass using lumen-apposing metal stents (LAMS) 4 . Early studies in malignant GOO suggest high success rates and favorable safety profiles 5 , but data on benign GOO remain limited. This multicenter study aimed to address this gap by evaluating the safety and efficacy of EUS-GE in patients with benign GOO, providing evidence to inform clinical practice.

## Patients and methods

This was an international, multicenter, retrospective cohort study conducted across 13 centers. All consecutive adult patients (18 years of age or older) who underwent EUS-GE for a benign etiology of GOO between November 2014 and November 2023 were included. Inclusion criteria required patients to have symptomatic GOO supported by cross-sectional imaging (computed tomography or magnetic resonance imaging) consistent with mechanical obstruction. Endoscopy databases or billing databases were queried at each center to identify patients. Using electronic medical records, the following data were recorded in a standardized form across centers: patient demographics, etiology of GOO, clinical symptoms including GOO scoring system, cross-sectional imaging of GOO, EUS-GE technical approaches, technical success, reasons for technical failure, procedure-related adverse events (AEs) with severity graded per the American Society of Gastrointestinal Endoscopy (ASGE) lexicon, procedure time, time to oral intake, diet tolerated after EUS-GE, GOO system score on follow up, endoscopic follow-up findings, recurrence of GOO and stent dysfunction, and total duration of follow-up. Patients were excluded if they had incomplete clinical or endoscopic data.

The primary endpoint was the rate of clinical success defined as the ability to tolerate solid food (grade 3) according to the GOO scoring system (0: no oral intake, 1: liquids only, 2: soft solids, 3: low residue or full diet). Secondary endpoints included rates of technical success, recurrence of GOO or stent dysfunction, and rate of AEs with the severity graded per the ASGE lexicon.Descriptive statistics were performed to summarize clinical, procedural, and follow-up characteristics. Continuous variables were reported as means with standard deviations (SDs) or medians with interquartile ranges (IQRs). Categorical variables consisting of the GOO symptom score (GOOSS) at baseline and post-procedure time points were compared using the chi-square test; *P* < 0.05 was considered statistically significant. All inferential tests were conducted with a significance level set at *P* < 0.05. Statistical analysis was performed using Stata/SE 17.0 (StataCorp, 2021).

## Results

A total of 72 patients, mean age 54.8 (SD +/- 17.8) with 42% females (n = 30), underwent EUS-GE for benign GOO indications. Etiologies of benign GOO in the study were diverse; surgical anastomotic stricture in 19% (n = 14), peptic stricture in 17% (n = 12), chronic pancreatitis in 15% (n = 11), acute pancreatitis in 13% (n = 9), superior mesenteric artery syndrome in 10% (n = 7), caustic stricture in 6% (n = 4), Crohn’s disease strictures in 3% (n=2), duodenal hematoma in 3% (n = 2), and others listed in Table 1 . Forty-six percent (n = 33) were evaluated to be nonsurgical candidates for management of GOO.

**Table:** **Table 1** Demographics and clinical characteristics of patients undergoing EUS-GE for benign GOO.

Clinical characteristics	N (%) or average (SD)
**Total participants**	72
**Age, mean (SD)**	54.8 (17.8)
Sex	
Female	30 (42%)
Male	42 (58%)
**Pre-BMI, mean (SD)**	21.6 (4.6)
**Benign indication for EUS-GE**	
Surgical anastomotic stricture	14 (19%)
Peptic stricture	12 (17%)
Chronic pancreatitis	11 (15%)
Acute pancreatitis	9 (13%)
Superior mesenteric artery syndrome	7 (10%)
Caustic stricture	4 (6%)
Duodenal stenosis	2 (3%)
Crohn's disease strictures in small bowel	2 (3%)
Duodenal hematoma	2 (3%)
Aortoduodenal syndrome	1 (1%)
Gastroparesis	1 (1%)
Pyloric stenosis	1 (1%)
Duodenal diverticulum	1 (1%)
Small bowel obstruction	1 (1%)
Extrinsic compression from liver tuberculoma	1 (1%)
Duodenal polyposis burden in setting of Gardner's syndrome	1 (1%)
Bouveret's syndrome	1 (1%)
Embedded stent in duodenal bulb	1 (1%)
**Surgical candidate for management of GOO?**	
Yes	33 (46%)
No	29 (40%)
Unknown	10 (14%)
**Total endoscopic follow up time, average in days (SD)**	417.8 (279.9)
**Longest total endoscopic follow up time, in days**	1113
**Total clinical follow up time, average in days (SD)**	426.3 (443.9)
**Longest total clinical follow up time, in days**	2145
BMI, body mass index; EUS-GE, endoscopic ultrasound-guided gastroenterostomy; GOO, gastric outlet obstruction; SD, standard deviation.	

The primary outcome, clinical success of EUS-GE for benign GOO, was assessed using the GOOSS. Pre-procedure, only 4% of patients (n = 3) were able to tolerate a solid food diet. Post-procedurally, this improved to 72% (n = 50) at 7 days, 82% (n = 56) at 30 days, and 90% (n = 57) at 60 days ( *P* < 0.00001). Mean time to resumption of oral intake was 20.1 hours (SD ± 14). Average follow-up time from initial procedure to last clinical follow up was 426.3 days (SD ± 443.9) and to last endoscopic follow up was 417.8 days (SD ± 279.9). The longest post-procedure follow-up extended over 5.8 years in the study.

Technical success was achieved in 97% (n = 70). Regarding EUS-GE technique in this cohort, the majority of endoscopists performed free-hand puncture technique (76%, n = 55) with AXIOS LAMS (97%, n = 70) at a size of 20 mm x 10 mm (59%, n = 40). Most commonly, endoscopists preferred to perform the procedure without dilation of the LAMS (71%, n = 51) or co-axial stent placement within the LAMS (96%, n = 69), displayed in Table 2 .

**Table:** **Table 2** Procedure characteristics and adverse events in patients undergoing EUS-GE for benign GOO.

Procedure/technical characteristics	N (%) or average (SD)
Technical success	70 (97%)
**Puncture technique style**	
Guidewire	12 (17%)
Direct puncture	55 (76%)
Balloon-assisted	5 (7%)
**LAMS type**	
AXIOS	70 (97%)
Spaxus	2 (3%)
**LAMS diameter and length**	
15 mm x 10 mm	23 (34%)
15 mm x 15 mm	5 (7%)
20 mm x 10 mm	40 (59%)
**Dilation post-LAMS deployment**	21 (29%)
**Co-axial stent placement post-LAMS deployment**	3 (4%)
**Adverse events**	N (%) or average (SD)
**Post-procedure adverse events**	
Early bleeding (< 48 hr)	2 (3%)
Late bleeding (> 48 hr)	1 (1%)
Early perforation (< 48 hr)	0 (0%)
Late perforation (> 48 hr)	0 (0%)
Early migration (< 48 hr)	1 (1%)
Late migration (> 48 hr)	3 (4%)
Other: gastrocolic fistula	1 (1%)
**Provider management of LAMS**	
Decided to replace LAMS at specified interval	14 (22%)
Decided to leave LAMS in place until clinical indication arises	39 (60%)
Removal of LAMS at follow up	12 (18%)
**Endoscopic findings at last endoscopic follow up**	
GE patent with stent in place	40 (68%)
GE not patent with stent in place	6 (10%)
Stent migration	1 (1.7%)
Stent removed	12 (20%)
Did stent occlusion ever occur?	11 (15%)
**Reasons for stent occlusion**	
Food debris	3 (27%)
Tissue overgrowth	5 (45%)
Blood clots	1 (9%)
Angulation	1 (9%)
Gastrocolonic fistula	1 (9%)
**Time to stent occlusion from initial procedure, median in days [IQR]**	213 [126–286]
**Was the stent ever upsized?**	3 (5%)
EUS-GE, endoscopic ultrasound-guided gastroenterostomy; GOO, gastric outlet obstruction; GE, gastroenterostomy; LAMS, lumen-opposing metal stent; SD, standard deviation.	

Rates of AEs during procedure were 6% (n = 4) with two stent misdeployments and two stent migrations/dislodgements. These AEs were all managed endoscopically with either removal of the LAMS and placement of an over-the-scope-clip (OTSC) over the gastric puncture site or repositioning of the LAMS endoscopically. Post-procedural AEs were low: most commonly stent occlusion in 15% (n = 11), early bleeding in 3% (n = 2), late bleeding in 1% (n = 1), early migration in 1% (n = 1), late migration in 4% (n = 3), and a gastrocolic fistula in 1% (n = 1), with all being categorized per the ASGE scoring system for AEs as mild. The patient with the gastrocolic fistula had removal of the LAMS followed by successful closure of the gastric puncture site with an OTSC and eventually underwent surgical revision for their benign GOO indication (anastomotic stricture).

Regarding provider management practices, the majority of endoscopists left the LAMS in place unless clinical indication occurred requiring reevaluation 60% (n = 39). Twenty-two percent of endoscopists 22% (n = 14) proactively elected to replace the LAMS at a median time of 4.5 months (IQR 3–6). Additionally, 18% (n = 12) removed the LAMS at endoscopic follow-up due to resolution of the clinical condition. At the last endoscopic follow-up, the stent remained patent and in place in 68% ( n =40). Recurrence of GOO symptoms occurred mostly due to stent occlusion in 15% (n = 11), tissue overgrowth in 45% (n = 5) or food debris in 27% (n = 3). There were no significant differences in clinical success, stent occlusion, or AEs between patients receiving 15 mm versus 20 mm LAMS. Stent occlusions were managed endoscopically (involving either removal of the occluded LAMS followed by replacement, or direct stent-in-stent placement depending on the clinical scenario and endoscopic findings). Median time to stent occlusion was 213 days (IQR 126–286).

## Discussion

Our international, multicenter, retrospective cohort study demonstrates that EUS- GE is a safe and effective treatment for benign GOO. These findings extend the growing body of evidence supporting EUS-GE as a minimally invasive alternative to surgery for GOO. The diversity of benign GOO etiologies in the study, including surgical anastomotic strictures, peptic strictures, chronic and acute pancreatitis, and superior mesenteric artery syndrome, highlights the broad applicability of EUS-GE.

The clinical success rate in our study was high, with the proportion of patients able to tolerate oral intake increasing from 4% before the procedure to 90% at 60 days post-procedure ( *P* < 0.00001). These results, coupled with our 97% technical success rate, align with previous studies of EUS-GE. A meta-analysis by Iqbal et al. found overall technical and clinical success rates of 92.9% and 90%, respectively, for EUS-GE across both malignant and benign etiologies 6 . Specifically focusing on benign GOO, Chen et al. reported similar technical success (96.2%) but a somewhat lower clinical success rate of 84% 7 . The aforementioned studies report AE rates of 9% and 16%, respectively, which are consistent with our favorable intraprocedural (6%) and post-procedural (10%) AE rates 6 7 . Similarly, Canakis et al. further supports our findings, demonstrating high technical (95%) and clinical (90.6%) success rates of EUS-GE for benign GOO, along with a favorable safety profile 8 .

Kahaleh et al. also showed that EUS-GE offers equivalent efficacy and safety for both benign and malignant etiologies, further validating its versatility across a range of clinical scenarios 9 .

In a single-center series by James et al., EUS-GE alleviated the need for surgery in over 80% of patients with benign GOO, with a low recurrence rate following stent removal, supporting its role as both definitive and bridge therapy 10 .

Comparisons of EUS-GE to surgical gastrojejunostomy have shown promising results. Khashab et al. found similar clinical success rates between EUS-GE and surgical gastrojejunostomy for malignant GOO (87% vs 90%, respectively), with a trend toward lower AEs in the EUS-GE group (16% vs 25%) 11 . Perez-Miranda et al. further compared EUS-GE to laparoscopic gastrojejunostomy, including some patients with benign etiologies 12 . They found significantly lower AE rates with EUS-GE (12% vs 41%, *P* = 0.0386), consistent with our findings. In addition, they reported shorter procedure times and lower costs associated with EUS-GE, highlighting its potential advantages over surgical approaches. Our study builds on these findings, suggesting that EUS-GE may offer a less invasive alternative to surgery with comparable efficacy and potentially improved safety.

The main advantages of EUS-GE for benign GOO include its minimally invasive nature, rapid resumption of oral intake, and potential for long-lasting symptom relief. However, limitations include the technical complexity of the procedure, requiring expertise in interventional EUS, and the lack of long-term follow-up data compared with established surgical techniques. Future research should focus on long-term outcomes of EUS-GE for benign GOO, direct prospective comparisons with surgical and other endoscopic approaches, and refinement of patient selection criteria to optimize outcomes. In addition, studies examining the learning curve for EUS-GE and strategies to improve technical success rates are warranted.

## Conclusions

In conclusion, our study demonstrates that EUS-GE is a safe and effective treatment for benign GOO, offering a minimally invasive alternative to surgery with high technical and clinical success rates and a favorable safety profile. As experience with this technique grows, EUS-GE may become an increasingly important option in management of benign GOO.
